# Improving the Uptake of Transcatheter Aortic Valve Replacement in Ontario

**DOI:** 10.7759/cureus.16364

**Published:** 2021-07-13

**Authors:** Abimbola K Saka, Joseph Ferenbok

**Affiliations:** 1 Institute of Medical Science, University of Toronto, Toronto, CAN; 2 Laboratory Medicine and Pathobiology, University of Toronto, Toronto, CAN

**Keywords:** tavr, tavi, uptake of tavr, aortic stenosis, transcatheter aortic valve repair, tavr uptake, implementing health innovation, barriers to innovation uptake, facilitators of innovation use in healthcare, surgical valve replacement

## Abstract

Background

The rising costs of healthcare delivery globally and the increasing research production rate create immense opportunities for implementing novel and more effective medical interventions that significantly benefit patient outcomes. However, the successful uptake of medical innovations is complex and often extremely contextual based on many sociopolitical and economic factors. These barriers to implementation can delay or derail new practices, procedures, products, and pharmaceuticals. Understanding the barriers to the successful implementation of medical innovations and the best practices and strategies to mitigate them is an extremely important area for translational research in health sciences.

This study examines the barriers and potential challenges in implementing medical innovations and the possible preemptive measures that can be addressed early to increase the use of life-saving medical innovations. We consider the importance of appropriate, timely, and user-defined implementation techniques as a critical component of the successful uptake of medical innovations and use the uptake of transcatheter valve replacement therapy (TAVR), which is an alternative life-saving intervention for patients at risk for surgical complications, in Ontario, Canada as the practical case study of the challenges and potential instructive opportunities to establish best practices for systematic and effective innovation uptake.

Methodology

In addition to contextual and informal investigations, a small pilot survey of decision-makers across the University of Toronto-affiliated teaching hospitals helped compare and contrast the barriers to medical innovation uptake (in the literature) with the suggested barriers to the successful implementation of TAVR. This study looks primarily at the role of funding, physician preference, clinical guidelines, and patient comorbidities as decision-making factors contributing to TAVR uptake. The study also explores how the differences and similarities of TAVR uptake related to the decision-making factors above can help develop recommended strategies to address future implementation barriers.

Results

We observed that the decision-makers across the surveyed institutions refer patients with intermediate to high risk for surgery for TAVR. Funding and physician preference were identified as possible barriers to TAVR uptake, with underlying comorbidities of patients being a primary decision determinant for TAVR referral. Physician preferences were based on multiple factors such as clinical judgment, patient comorbidities, clinical guidelines, knowledge, TAVR, and surgical valve replacement skills.

Conclusions

To the best of our knowledge, this study is one of the first to use the Toronto Translational Thinking Framework to assess an innovative treatment uptake in the Ontario healthcare system. Although the study sample size was 11 and did not reflect the views of all decision-makers regarding TAVR use in Ontario, the survey reflected participants who directly make decisions regarding TAVR use, strengthening the credibility of the survey results. The insights from this study are intended to inform both the continued implementation of TAVR and to contribute to a broader field of investigation that aims to identify and operationalize the principles and best practices of translational research that may contribute to the efficacy of implementing other medical innovations in Ontario hospitals and beyond.

## Introduction

Innovation in medical care delivery to cope with rising healthcare costs; an aging population; the tidal wave of new technologies, products, and techniques; and the need to improve the safety and quality of care for patients is a major priority globally. In these respects, innovation offers new and improved approaches to diagnosing, treating, and managing diseases. However, because of the barriers to medical innovation uptake, it is not always easy to reap the benefits of innovations.

While innovations are attempted widely in healthcare, most health innovation ideas do not progress into viable healthcare delivery changes. Less than 5% of drug or technology innovations become successfully implemented and sustained [1]. Only a few successfully developed and pilot-tested innovations are implemented effectively, achieve their expected outcomes, and eventually become institutionalized into standard practices. The analysis of the various strategies to facilitate the uptake of innovations provides better insight into why the uptake of medical innovations is low.

The paradox of innovation is that institutions that have been the most successful often think they have more to lose from change. Because their past practices have been effective, they want to retain them, indirectly impeding the uptake of innovations [2]. In addition to institutional factors, systemic policies, patient culture and values, funding availability, and physician preference are identified barriers that may impede any innovation uptake [3].

Several active efforts to integrate new clinical innovations into routine practice settings have been established. An example of this is incorporating medical innovations into clinical guidelines to facilitate uptake and ensure clinicians adhere to best care practices.

Pathophysiology of aortic stenosis

The aortic valve is one of four valves in the heart, which allows the outflow of oxygenated blood from the heart to the vital organs and ensures adequate circulation to the body. The severity of the aortic valve’s narrowing is progressive, and if left untreated, it can lead to death (mostly from heart failure). The definitive treatment is to remove the valve and replace it with an artificial valve, which often involves open-heart surgery [4,5]. Patients at risk of developing complications from surgery (such as blood loss) are regularly monitored for symptoms and are treated based on the symptoms they present with or with a balloon to open the narrowed valve. Aortic stenosis can occur due to a congenital defect, infections such as rheumatic fever, or calcium deposition around the valve due to aging. In western countries and individuals aged 65 and above, aortic stenosis is mostly due to calcium accumulation in the valve, known as degenerative calcification of the aortic valve (the most common indication for valve replacement).

Context

In Canada, the prevalence rates of all valvular diseases are 2.5% at age 75 and 0.4% at age 80 [5]. However, for aortic stenosis, representing the most common form of valvular disease, the prevalence rates are 2.0% at age 75 and 8.1% at age 85 [6]. Studies have documented transcatheter aortic valve replacement (TAVR) as a safe and effective alternative to surgical aortic valve replacement (SAVR) in high-, intermediate-, and low-risk patients because of its benefit for patient outcomes and reduction in mortality rate [7]. The 30-day and one-year mortality rates after valvular replacement have been reported to be lower in individuals undergoing TAVR across all the risk categories, with the most significant decrease noted in high- and intermediate-risk groups [8].

After TAVR was introduced in 2011 and its implementation in California hospitals, there was a decrease in surgical valve replacements in the United States [9]. The reduction in the SAVR rate was associated with the rapid uptake of TAVR in high-risk surgical patients. TAVR cases have gradually increased, rising from 224 cases in 2011 to 1,022 cases in 2018 across Ontario institutions. However, compared with the old standard of care (SAVR), there is still a significant variation in the proportion of patients undergoing SAVR compared with TAVR, with 1,720 cases in 2011 and 1,978 cases in 2018 [10]. The observed change in the rate of TAVR cases performed can be attributed to several factors impacting its implementation for use, such as funding and new evidence supporting the impact of TAVR on patient outcomes.

Until 2012, in Ontario, TAVR was funded through Health Canada’s Compassionate Approval, and hospitals could develop and establish TAVR implementation programs using funds from global budgets, research, and foundation grants. However, after Health Canada approved TAVR for use as an alternative to SAVR and distribution in the Canadian market, it has now become an insured service across Ontario hospitals through the Ministry’s global budgets [11].

Compared with the more invasive and older standard of care (SAVR), TAVR is a minimally invasive procedure used as an alternative to treat patients with severe aortic stenosis because of the risks of complications associated with surgery [12]. The incidence of aortic stenosis increases with age, with about 29% of cases occurring in individuals aged 65 and older and about 37% occurring in individuals aged 75+ [12]. Known for its poor prognosis, aortic stenosis has a one-year survival rate of about 60% and a five-year rate of about 32% [12].

The recommended intervention for managing severe aortic stenosis in moderate to high-risk patients in the American Heart Association/American College of Cardiology guidelines is TAVR [12]. This recommendation is based on multiple factors, such as surgical risk, patient fragility, comorbid conditions, and patient preferences and values. Recent clinical trials have also shown that TAVR is not inferior and may be superior to SAVR in reducing mortality, stroke, and rehospitalization. This indicates substantial benefits for patients [12,13]. This paper explores how the differences and similarities of TAVR uptake related to decision-making factors can help develop recommended strategies to address future implementation barriers.

## Materials and methods

**Figure 1:**
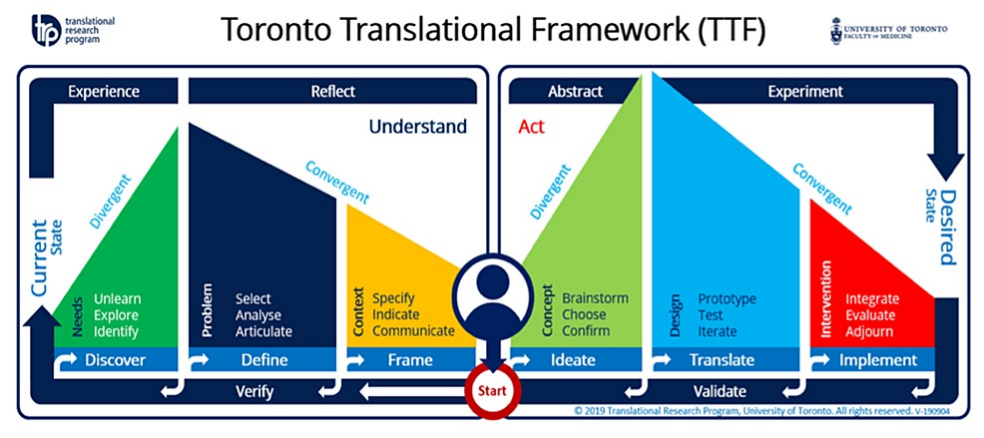
Toronto Translational Thinking Framework.

**Figure 2:**
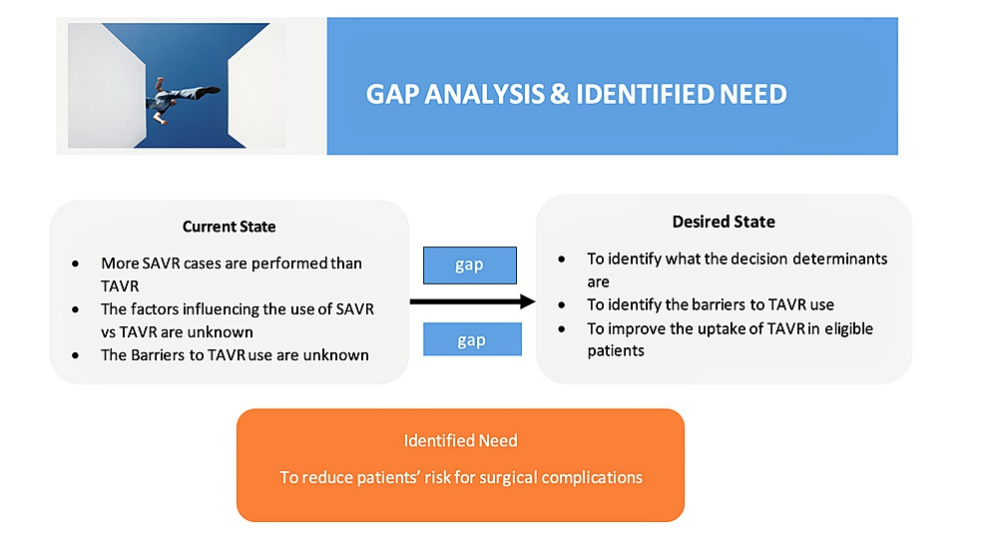
Gap analysis and identified need. SAVR: surgical aortic valve replacement; TAVR: transcatheter valve replacement therapy

Using the Toronto Translational Thinking Framework (Figure 1), we explored the current state, the desired state, and the need for severe aortic stenosis treatment. A gap analysis (Figure 2) was conducted via informal interviews of interventional cardiologists and phone calls to multiple Ontario hospitals, such as Kingston General, St. Mary’s Hospital, St. Michael’s Hospital, and Trillium Health Partners.

The gap analysis showed that, despite the benefits of TAVR over SAVR in patients with severe aortic stenosis and intermediate and high risks for surgical complications, there is variation in the number of SAVR cases versus TAVR cases being performed across hospitals in Ontario. From the gap analysis, we also identified that the barriers to TAVR use and the factors influencing use were unknown; however, the “identified need” was to reduce patients’ risks for surgical complications. To further understand the principal reasons for this variation, identify the barriers to TAVR use, and develop strategies to address potential barriers, we conducted a cross-sectional survey of decision-makers across the University of Toronto-affiliated hospitals using a validated electronic survey [14].

**Figure 3:**
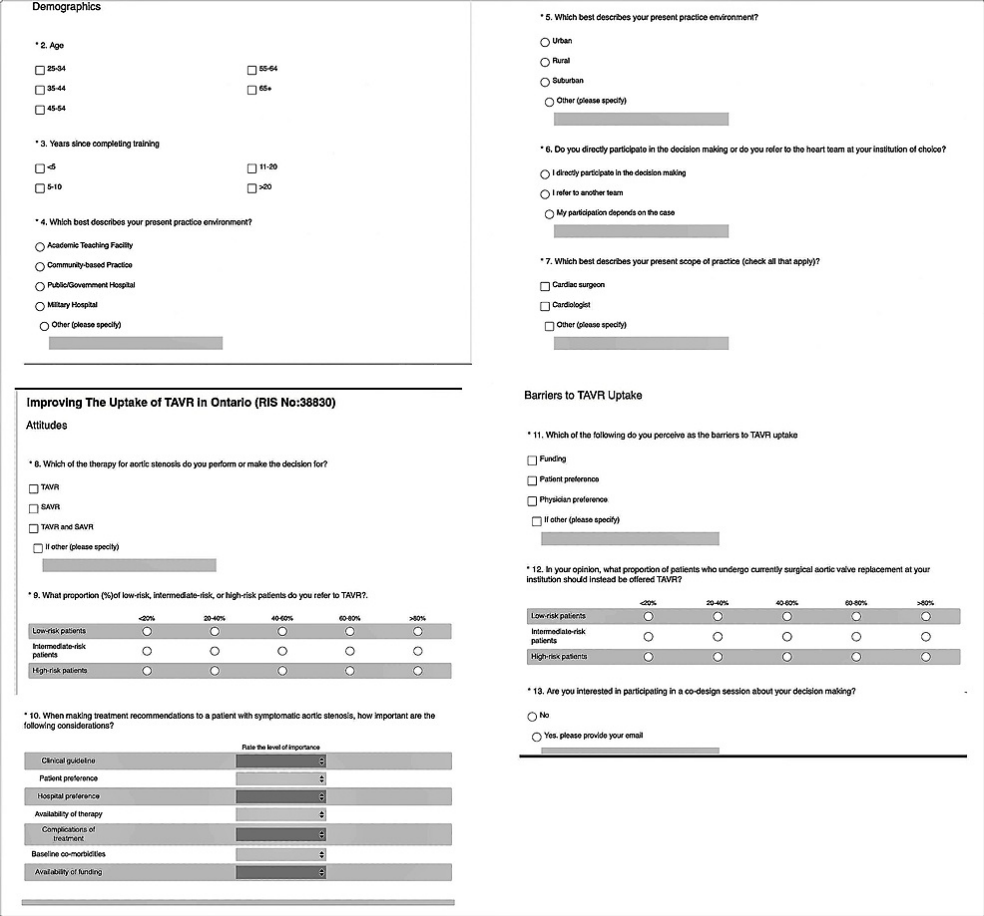
TAVR survey questions. TAVR: transcatheter aortic valve replacement

Participants were identified through a partner organization’s healthcare provider database, and 43 decision-makers were recruited to complete the survey. Of the 43 recruited participants, 11 completed responses were obtained, giving a 25% response rate. The electronic survey (Figure 3) inquired about the characteristics of the decision-makers, their knowledge of the procedure, their behaviors regarding the use of TAVR and SAVR, and the aspects of their decision-making process for referral to SAVR or TAVR. Given the study’s timeline, we focused on University of Toronto-affiliated institutions, with a predicted sample size of 43. Only individuals who self-selected as decision-makers for TAVR or SAVR were included in the study. Individuals who were not involved in the decision-making for SAVR or TAVR were excluded from the study.

All data obtained from the participants were anonymized and stored securely in a password-protected database. The survey responses were then analyzed using the Statistical Package for the Social Sciences (IBM Corp., Armonk, NY) to conduct descriptive analysis.

## Results

In our analysis of the survey results, the primary factors influencing the decision to opt for either TAVR or SAVR were clinical guidelines and baseline comorbidities. In addition, funding and physician preference appeared to be the primary barriers to TAVR uptake. Below, we further discuss the differences and similarities of TAVR uptake related to decision-making factors.

Barriers to innovation uptake in healthcare

Several barriers are known to impede the uptake of innovations in healthcare. The key identified barriers to innovation uptake are described below.

Funding

It is critical to promote successful implementation at the early stage of an innovation process. Lessons from various studies have shown that funded innovations can only have a transformative impact on lives and the economy if funding as a potential barrier is identified at the early stage of innovation. In many healthcare situations, limited resources attributed to a lack of funding have been identified as a barrier to promoting the buy-in of innovation from decision-makers. Desveaux et al. [15] highlighted funding as a key barrier to innovation uptake. They clarified the need to tailor incentives and rewards to the adopters’ interests and values. In cases where this was done, high uptake and use of medical inventions were noted. The high uptake was attributed to adopters’ intrinsic motivation to use new programs or treatments. From the study of Desveaux et al. [15], it is evident that funding that is not directly secured for continued implementation directly affects sustainability and innovation uptake.

**Figure 4:**
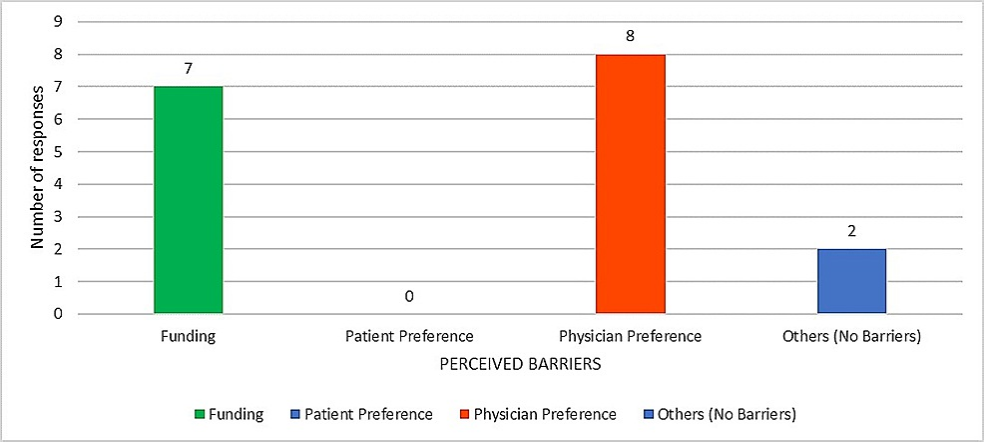
Perceived barriers to TAVR uptake. This bar chart shows the total number of participants who selected funding, patient preference, physician preference, and others (in this case, the participant indicates no perceived barrier) as the perceived barriers to TAVR uptake. The color-coding in the chart is as follows: blue shows the number of participants who selected others as the barrier to TAVR uptake (in this case, the participants entered no barriers). Red indicates the number of participants who selected physician preference as a barrier, and green indicates the number of selected funding participants. From the results obtained, it appears that physician preference and funding are the primary barriers to uptake. Patient preference was not indicated as a perceived barrier to TAVR uptake. TAVR: transcatheter aortic valve replacement

The results of the studies mentioned above are consistent with the case of TAVR, where a change in the funding structure (from Health Canada’s Compassionate Approval to the Ministry’s Global Budget funding) affected the number of TAVR cases and hospitals implementing TAVR in Ontario [11,16]. However, despite a direct funding source from the Ministry, our survey showed that funding remains a barrier (Figure 4). The possible reasons for this observation and the strategies to address this barrier are discussed below.

Institutional and Systemic Policies

A body of literature shows that, until there is a shift in the culture within an organization, it is beneficial to focus solely on healthcare providers who may likely use an innovation, leaving providers who are not ready for the change to do so later [17-19]. It is also important to seek key opinion leaders internally in an organization and external to the organization to champion innovation; this can help develop policies that will facilitate successful implementation [18]. In addition, creating an environment that allows the co-creation of innovation and collaboration among stakeholders and users to improve patient and economic outcomes will promote successful innovation uptake [18]. From the analysis of our study, institutional and systemic policies do not appear to be barriers to TAVR use.

Patient Cultures and Values

**Figure 5:**
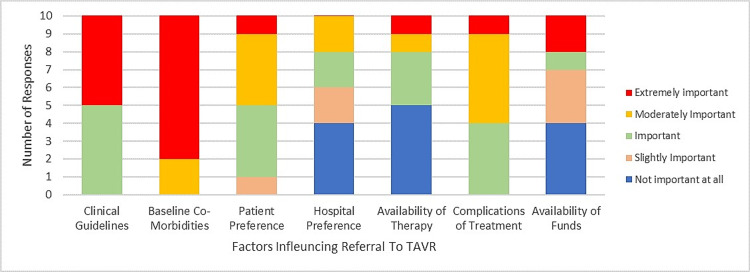
Factors influencing the decision to treat with TAVR or SAVR. This bar chart shows how participants rated clinical guidelines, hospital preference, availability of therapy, complications of treatment, baseline comorbidities, and the availability of funds in their decision-making to use either TAVR or SAVR. Red indicates that the factors are extremely important in decision-making. Yellow demonstrates that the factors are moderately important. Green indicates that the factors are important. Orange indicates that the factors are slightly important when deciding which therapy to use. Baseline comorbidities and clinical guidelines appear to be the primary factors influencing the decision to treat with either SAVR or TAVR. SAVR: surgical aortic valve replacement; TAVR: transcatheter valve replacement therapy

A study conducted in 2015 by Bulger [18] in the United States emphasized the importance of culture and value norms in healthcare and how it affects an intervention. There is a need to integrate patients’ perspectives, values, and needs when developing an intervention or recommending treatment for a disease condition; this need guided the decision to include the “culture of health” to ensure patient perspectives are prioritized when deciding on a treatment, as this will affect the innovation uptake [18]. As shown in our results, the patient barrier does not appear to be a factor impeding TAVR uptake, and patients’ beliefs and values are important factors when deciding on TAVR use. This is likely because the implementation strategy used for facilitating TAVR uptake is the clinical guideline that outlines the need to consider patient values when deciding between TAVR and SAVR (Figure 5).

Physician Preference

With the knowledge of the impact of physician preference on the uptake and sustainability of innovations, Shbool et al. [20] outlined the importance of value analysis involving a cross-functional team of clinicians, value analysis professionals, supply chain professionals, and administrators to discuss the value of implementing innovation in any organization. According to these researchers, a misalignment of physician preference and organizational priorities relating to an invention affects the use and uptake of new interventions. The failure to consider physician preference when securing an innovation may imply that physicians will leave the organization or become demotivated [20].

Similar to the study by Shbool et al. [20], in our study, physician preference appeared to be a major barrier to TAVR uptake. The observed barrier attributed to physician preference seen in the case of TAVR may indicate some reluctance to move away from a more traditional treatment (surgery), particularly considering that most participants in our study finished their training more than 20 years ago (Table 1). To address this problem, it may be necessary for implementation leads at each organization that is eligible to treat patients with TAVR to provide continued medical education and regular updates on TAVR to physician users.

**Table 1:** Years since training and decision-making for TAVR. Participants were asked about the number of years since training completion and whether they participated in direct decision-making regarding TAVR. The number of participants for each time category is cross-tabulated with whether they participated in direct decision-making. TAVR: transcatheter valve replacement therapy

		Decision-making on TAVR	
		Yes	No
Years since completing training	<5 years	1	0
	5–10 years	0	0
	11–20 years	2	1
	>20 years	7	0

## Discussion

Transcatheter valve replacement therapy implementation and uptake in Ontario

Like most contributions to the body of literature on medical innovations that focus on improving patient outcomes and reducing healthcare expenditure, TAVR is a novel intervention that can improve health outcomes for more patients and improve long-term health expenditure with successful implementation [16]. As seen in the studies discussed in this paper, some of the highlighted barriers to innovation uptake are present in the TAVR case. The study survey focused on the decision-makers (healthcare providers) for aortic stenosis treatment (SAVR and TAVR).

In TAVR, funding and physician preference were the most selected barriers to TAVR uptake across the surveyed academic institutions (Figure 3). The healthcare providers indicated that the primary determinants for TAVR use were clinical guidelines and patients’ underlying comorbidities (Figure 4). The secondary determinants included patient preference and risks for procedural complications (Figure 4). Therefore, it is evident that integrating TAVR into the clinical guideline, an effective strategy outlined in the Expert Recommendations for Implementing Change compilation (ERIC), is the primary facilitator for use. However, this alone is not effective in promoting its use over open-heart surgery (SAVR), which is evident in the gradual increase in the rates of TAVR versus SAVR cases performed across Ontario institutions (Table 2).

**Table 2:** TAVR and SAVR procedures performed during 2011/2012 and 2017/2018 across Ontario institutions. SAVR: surgical aortic valve replacement; TAVR: transcatheter aortic valve replacement Data retrieved from Corhealth Ontario, 2018 [10].

Procedure	2011/2012	2012/2013	2013/2014	2014/2015	2015/2016	2016/2017	2017/2018
SAVR	1,720	1,691	1,843	1,764	1,864	1,887	1,978
TAVR	224	341	486	646	745	863	1,022

It is crucial to develop targeted strategies that can promote TAVR use. An example of such an approach could be relationship-building among institutions to co-create and identify more champions at each organization to facilitate uptake.

In 2016, considering both the economic value and patient outcome of TAVR, the Ontario Health Technology Advisory Committee recommended that TAVR be publicly funded in select hospitals, as determined by CorHealth Ontario. CorHealth Ontario advocates for safe, patient-centered, and equitable services in Ontario and focuses on the three following domains: cardiac care, stroke, and vascular care. The recommendation to fund TAVR was based on the finding that it is minimally invasive and does not result in higher mortality than SAVR, with the knowledge that both therapies have comparable benefits in improving patients’ quality of life within the first year after valve replacement [16,21].

The health technology assessment study conducted by Health Quality Ontario in 2020 on the cost analysis of TAVR and SAVR showed that TAVR is more expensive, but on average, more effective than SAVR, with TAVR producing more quality-adjusted life-years [21]. In the analysis, patients reported that TAVR is less invasive and results in a substantial reduction in physical and psychological effects after the procedure, thereby improving their quality of life. Ontario’s net budget impact on public funding was estimated at $2-$3 million each year for the next five years, meaning that funding is potentially a significant barrier to TAVR uptake and implementation across Ontario’s institutions [16,21]. Thus, it is not surprising that financial costs are perceived as barriers to TAVR uptake.

While we can explain funding as a barrier to TAVR use by examining the available funding of hospitals on a case-by-case basis, the barrier of preference may depend on the referring physician. Using a strategy founded on a robust theoretical framework (discussed in this paper), through active engagements with the key stakeholders; collaboration between physicians, institutions, and the government; and knowledge dissemination, can enhance this innovative treatment’s uptake to improve more lives.

Strategies to address barriers to innovation uptake

Frameworks and Tools

Several implementation frameworks have been established to identify potential barriers that may impede the use of medical innovations. Examples of these frameworks are discussed below.

The Consolidated Framework for Implementation Research (CFIR): The CFIR is a menu of constructs across five domains known to facilitate a successful implementation (Table 3). The CFIR constructs reflect both Rogers’s Diffusion of Innovation theory and Greenhalgh et al.’s compilation of implementation strategies. It serves as a practical guide for systematically assessing the barriers and facilitators of successfully implementing an innovation [22].

ExpandNet/World Health Organization (WHO) Framework: The ExpandNet/WHO framework constitutes nine steps for developing a scaling-up strategy. This approach assesses an innovation’s potential to be successfully implemented. The ExpandNet/WHO framework guides users through a thorough analysis of the problem and develops recommendations for actions that will form the basis for improving the uptake of an implemented innovation [23].

Nose to Tail Tool (NTT): The NTT offers innovation teams, consisting of innovators and essential stakeholders, including end-users and decision-makers, and a guide to identifying critical considerations from all the stakeholders’ perspectives that should be addressed at each stage of the innovative process. Furthermore, it helps identify contextual obstacles at each stage that may impede the successful implementation of an innovation. (These barriers need to be addressed and overcome at each innovation stage.) The NTT is an iterative tool that enables stakeholders to facilitate successful innovation [24].

Promoting Action on Research Implementation in Health Services (PARIHS) Framework: The PARIHS framework provides a way to implement innovation in practice by examining three key elements for knowledge translation. According to this framework, successful implementation and use depend on three factors: evidence supporting that the invention works, the context in which the proposed invention is to be implemented, and facilitators that will promote uptake [25].

Implementation for Change Framework: Similar to the PARIHS framework, the Implementation for Change Framework considers the barriers and facilitators within an institutional context, from the organizational leadership to administrative management, decision-makers, and the users of innovation [26]. Some examples of techniques used in ERIC as implementation strategies are shown in Table 3.

**Table 3:** Examples of strategies using ERIC. Adapted from Powell et al. [28]. Expert Recommendations for Implementing Change

Implementation strategy	Activities
Relationship-building strategy	Identify and prepare adopters and champions at each institution
Develop an open resource sharing agreement to identify all patients in need of the innovation	Develop partnerships across all institutions with the invention to ensure access to resources needed for implementation
Conduct ongoing training for clinicians (i.e., residents, fellows, and attending physicians)	Plan and conduct continuous clinical training for clinicians using the intervention
Obtain interactive feedback through facilitation	Develop a process of interactive problem-solving
Utilize financial strategies	Fund and develop financial incentives to adopt an innovation through contracting processes that will motivate healthcare providers to recommend the innovation
Engage end-users	Engage patients and providers as active participants
Support clinicians	Create new clinical teams and revise professional roles to eliminate service barriers to care and personal policies

Implementation Strategies

The identified implementation strategies are described below.

Rogers’s Diffusion of Innovation: Diffusion of Innovation theory represents one of the best strategies to address barriers to implementing innovations. It describes the diffusion of innovation as moving from innovation to the decision to minimize uncertainty about the invention. It involves steps on how individuals or decision-makers adopt or reject a design. These steps are knowledge, persuasion, decision, implementation, and confirmation [27].

Expert Recommendations for Implementing Change: The ERIC is a compilation of 73 well-researched implementation strategies to address barriers to innovation uptake [28].

Lessons Learned from Transcatheter Aortic Valve Replacement Uptake

Despite the clear benefit of TAVR for patients with severe aortic stenosis, some key challenges persist. Funding and physician preference are the main barriers identified in this study, a finding shared by previous studies discussed in this paper. Previously identified facilitators to implementation include the early involvement of key stakeholders in the innovation process, as outlined in the ERIC strategies.

**Figure 6:**
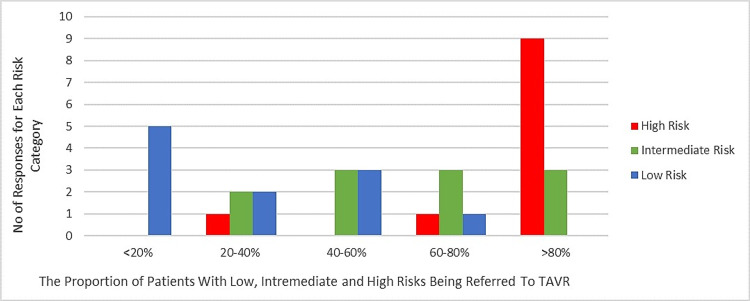
Sampled responses for the proportion of patients currently undergoing TAVR. This bar chart shows the proportion of low-, intermediate-, and high-risk patients referred to TAVR by the survey participants. Blue indicates patients with low risk, green indicates patients with intermediate risk, and red indicates patients with high risk. This bar chart shows that most of the survey participants (9/11) refer a high proportion of high-risk patients (>80%) to TAVR. TAVR: transcatheter valve replacement therapy

**Figure 7:**
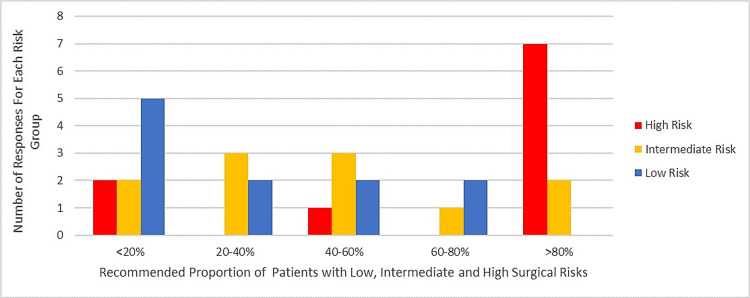
Sampled proportion of patients currently undergoing SAVR who should be offered TAVR. This bar chart shows the proportion of low-, intermediate-, and high-risk patients presently treated with surgery who should be offered TAVR instead. Blue shows the number of responses for the proportion of patients with low risk. Yellow shows the number of responses for the proportion of patients with intermediate risk, and red shows the number of responses for high-risk patients. From this bar chart, our analysis suggests that there is a consensus (7/11 participants) that >80% of patients with high risk currently undergoing SAVR should be recommended to undergo TAVR. SAVR: surgical aortic valve replacement; TAVR: transcatheter valve replacement therapy

From our case, we learned that there is a discrepancy between the proportion of patients who are currently being referred to TAVR (Figure 6) and the proportion of patients that physicians would like to recommend TAVR (Figure 7). Physicians would like to refer >80% of patients who presently undergo SAVR to TAVR (particularly those with high and intermediate risks). The reason for this observed discrepancy can be explained by the perceived barriers to TAVR uptake (Figure 4). We also established that having a sustainable funding structure is not sufficient to remove funding as a barrier or promote innovation uptake. Our observation may imply that, although there is ongoing funding allocation by the Ontario Ministry of Health and Long-Term Care, the allocated amount is not sufficient to cover all cases of intermediate to high-risk patients; thus, only those with significant comorbidities and risks for complications are recommended for TAVR at each institution. To address this issue, implementation team leads at each institution should work collaboratively to strengthen their relationships with the Ministry by providing supportive evidence that can help convince the Ministry to increase the allocated funds for TAVR procedures. This is an adapted strategy from the CFIR implementation framework and the ERIC compilation of implementation strategies.

## Conclusions

To the best of our knowledge, this study is one of the first to use the Toronto Translational Thinking Framework to assess innovative treatment uptake in the Ontario healthcare system. Although the TAVR study sample comprised 11 participants and did not reflect the views of all decision-makers regarding TAVR use in Ontario, the survey reflects participants who directly make decisions regarding TAVR use, strengthening the credibility of the survey results. It may also be relevant to address physician perspectives on using TAVR for the recommended patient group by conducting a province-wide system process improvement workshop to help understand physician perspectives and identify potential ways to maximize current resources through collaboration among implementation leads at each institution, organizations’ internal and external key opinion leaders, and physicians deciding on TAVR to optimize TAVR use in eligible patients and remove the identified barriers to uptake.

Effective implementation practices used to address barriers to innovation uptake are those that constitute early involvement of key stakeholders in the innovation process, practical training of users of the innovation, early identification of system enablers, alignment of systemic policies and culture to facilitate an openness to change, allow early integration of physician and patient perspectives and cultural views in the innovation process, involve key opinion leaders and champions within an organization, afford early adoption of measures to reduce administrative burden, and include a collaborative funding effort between teams to secure ongoing funding and reduce cost. Adopting these practices may help improve the future uptake of medical innovation use.
